# Empowering Communities During the COVID-19 Pandemic Through Mothers’ Support Groups: Evidence From a Community Engagement Initiative in Sri Lanka

**DOI:** 10.9745/GHSP-D-22-00402

**Published:** 2023-04-28

**Authors:** MSD Wijesinghe, BMI Gunawardana, WMPC Weerasinghe, SASC Karunarathne, VCN Vithana, RMNU Rajapaksha, R Batuwanthudawe, RPP Karunapema

**Affiliations:** a Health Promotion Bureau, Colombo, Sri Lanka.

## Abstract

Community empowerment has previously been used to mitigate the effects of health emergencies, such as outbreaks of Ebola virus disease and Zika virus. In Sri Lanka, mothers’ support groups (MSGs) aim to promote community health, well-being, and nutrition practices through community engagement. With the emergence of the COVID-19 pandemic, MSGs were mobilized by the Health Promotion Bureau in response to the pandemic. Key activities MSGs engaged in during the COVID-19 response included establishing communication networks, creating a supportive environment for preventive behaviors, organizing vaccination clinics, distributing essential food and medicine, organizing recreational activities, promoting home gardening, and monitoring community activities. We examine how these community-based empowerment initiatives successfully assisted in the pandemic response. Health officials can help to build more resilient communities that are better prepared for future health emergencies by valuing the community-level methods used in overcoming COVID-19 and further strengthening the skills of community group members.

## Abstract (precis)

Community empowerment activities conducted by mothers’ support groups in Sri Lanka played a critical role in COVID-19 outbreak control among communities.

## Teaser

**Key Findings:**

- During the COVID-19 response, mothers’ support groups (MSGs) aimed to promote community health and well-being through community engagement activities, including sharing information, education, and communication materials about COVID-19; creating a supportive environment for infection prevention and control practices; distributing essential food items and medicine to people in need; and organizing recreational and social support activities.
- A key strength of using MSGs was the well-established trust and relationships within communities and their direct access to communities. Other facilitating factors were the support received from existing community partnerships and training and capacity-building from district health education officer networks.
- Key barriers to conducting these activities included reluctance of public health workers to work together with MSGs, competing stakeholder agendas, and lack of support for appreciation of MSG activities from local health authorities.

**Key Implications:**

- Community empowerment can play an essential role in disease outbreak control activities, and its potential should be further studied.
- Well-implemented community engagement strategies can support health systems in overcoming pandemics.
- Health officials can strengthen the skills of community group members to help build more resilient communities during future pandemics.

## INTRODUCTION

Community empowerment is a key concept of health promotion and is widely practiced worldwide. It was well documented in the Ottawa Charter, the first international conference on health promotion,^1^ and its value was re-emphasized in the Jakarta Declaration on health promotion. An empowered community has been identified as one of the major goals that can be achieved through health promotion.^2^

As defined by the World Health Organization, community empowerment is a process of enabling communities to increase control over their lives.^3^ It is not an easy task and requires the use of multiple strategies and activities. Community engagement, one of the major strategies that can be used for community empowerment, involves engaging community members in different ways and to differing degrees, classifiable into 3 broad categories^4^:

1. Community members or voluntary organizations working in partnership with formal organizations when deciding and implementing interventions
2. Members of voluntary and community organizations getting things done; fostering community links; and building skills, self-esteem, and networks
3. Informally engaging with the community, involving social support mechanisms based on kinship, friendship, and neighborhood networks

The use of community engagement in disease response is not a novel idea. It has been adopted and known to be a key component in responses to past epidemics of Ebola virus disease, Zika virus, Middle East respiratory syndrome, and severe acute respiratory syndrome. Facilitators of and barriers to the success of community engagement in response to disease outbreaks are listed in Table 1.

**TABLE 1.** Key Facilitators and Barriers^5^ to Successful Community Engagement During Pandemics

**Facilitators**	**Barriers**
Engagement at the early stage	Lack of understanding about the context
Ability to adjust an ongoing process to cater to a new need	Choosing inappropriate actors to engage
Decentralization of the management	Poor trust in the state, its organization, and media channels
Facilitation of multisectoral engagement	Inconsistency of the messages
Assignment of clear roles and sharing responsibilities with every stakeholder	Poor allocation of responsibilities
Maintenance of a continuous connection with the ongoing community level response	Insufficient training and support
Use of preexisting actors appropriately	Inadequate resources
Use of clear 2-way channels for communication	Weak health infrastructure
	Contextual challenges

## THE COMMUNITY-BASED APPROACH TO COVID-19 PREVENTION

Communities share a common interest irrespective of their unique connections. Enabling communities to have more control over their lives through empowerment is an essential aspect in the context of health promotion.^6^ An empowered community can be mobilized during a health crisis and is very useful in fighting pandemics.^5^ It can support the state response in various ways, such as supporting information dissemination, fundraising for economic support, identifying the most vulnerable people (and helping them), and improving public compliance to preventive actions.^7^

On January 30, 2020, the World Health Organization declared COVID-19 a Public Health Emergency of International Concern, and it spread to worldwide within a short period of time.^8^ The situation was more complicated during the initial phases of the pandemic when there was no vaccine available. Personal protective equipment and good health hygiene were the only preventive methods during these initial phases. Therefore, a greater need existed for behavior change communication through community engagement, which many countries have been using and benefiting from since the beginning of the outbreak.

## THE MOTHERS’ SUPPORT GROUP APPROACH IN SRI LANKA

The global history of mothers’ support groups (MSGs) began in the 1950s.^9^ In 2002, the MSG concept was introduced in the Hambanthota district in Sri Lanka with guidance from the Medical Officer of Health in Sooriyawewa.^10^ Most MSGs in other countries function based on the concept of the “experienced mother helping the new mother.” However, presently, in Sri Lanka, MSGs aim to promote health, well-being, and nutrition practices in the community through community engagement.

In Sri Lanka, MSGs aim to promote health, well-being, and nutrition practices through community engagement.

Each MSG comprises 5–20 participants from families with children aged younger than 5 years. MSGs seek to improve nutrition-related practices in the communities by being change agents; improve family health in the community by enhancing awareness and practices; encourage the community in local income generation; prevent substance abuse in the community; support early childcare and development; improve parenting skills; contribute to optimizing environmental health; and address any priority health needs within the local communities.^10^

Sri Lanka is divided into 26 health administrative districts, and each district is further subdivided into several medical officer of health areas. Within each of these areas, there are further small subdivisions called public health midwife (PHM) areas. Within each PHM area, several MSGs function to improve the health and well-being of people at the village level.^10^ These community-based approaches have been used for many health-related activities by the Ministry of Health. The Health Promotion Bureau (HPB) of Sri Lanka is the national focal point for monitoring all the activities of the MSGs. In 2015, the HPB published a guideline on MSG activities, and it was further edited and republished in 2018.^10^ A 2014 review of the functioning of MSGs in the Northern and Eastern Provinces of Sri Lanka reported that 64% of the MSGs assessed spent nearly 14 hours on activities per month.^11^ Another study by Gamagedara et al. found that the contribution of MSGs (by creating awareness and health education) to children’s attendance at growth monitoring programs and feeding during illness was statistically significantly higher than those who were not involved in MSG activities.^12^ MSGs are also not the only community-based program functioning at the village level in Sri Lanka. Many other community-based organizations, such as the Sarvodaya movement, operate in parallel with MSGs in some areas. However, most of them work synergistically with MSGs.

Table 2 shows the total number of functioning MSGs in each district and the number of MSGs per 10,000 population as of 2021 as reported to the Reproductive Health Management Information System run by the Family Health Bureau in Sri Lanka.^13^ Currently, the Reproductive Health Management Information System only captures data from functioning MSGs. Therefore, the data regarding nonfunctioning MSGs are minimal.

**TABLE 2.** Number of Functioning Mothers’ Support Groups in Each District of Sri Lanka as of 2021 (per 10,000 Population)

Province	District	District Population^14^	Number of Functioning MSGs^13^	MSGs per 10,000 Population
Western	Colombo	2,462,428	261	1.06
	Gampaha	2,428,443	128	0.53
	Kalutara	1,289,543	290	2.25
Southern	Galle	1,140,247	600	5.26
	Matara	869,753	414	4.76
	Hambanthota	674,330	369	5.47
Uva	Badulla	892,068	229	2.57
	Monaragala	505,231	156	3.09
Central	Kandy	1,490,535	353	2.37
	Mathale	527,301	248	4.70
	Nuwara Eliya	778,212	308	3.96
Sabaragamuwa	Rathnapura	1,187,617	40	0.34
	Kegalle	894,609	162	1.81
North Central	Anuradhapura	948,767	249	2.62
	Polonnaruwa	445,896	99	2.22
North Western	Kurunegala	1,732,982	335	1.93
	Puttalam	842,328	81	0.96
Eastern	Ampara	285,574	118	4.13
	Kalmunai	459,048	144	3.14
	Trincomalee	436,474	135	3.09
	Batticaloa	583,035	177	3.04
Northern	Jaffna	624,950	203	3.25
	Kilinochchi	132,091	66	5.0
	Mannar	113,379	71	6.26
	Mullaitivu	98,569	62	6.29
	Vavuniya	193,279	71	3.67
Total		22,036,689	5369	2.44

Abbreviation: MSG, mothers’ support group.

From the early phase of the pandemic until the present, the HPB used the MSG approach in communities to conduct community empowerment activities. In this article, we review the methods that MSGs used to empower communities in overcoming the COVID-19 pandemic.

## EVALUATING THE COMMUNITY-LEVEL METHODS FOR OVERCOMING COVID-19

At the beginning of 2020, the HPB sent generic strategies to all MSGs to empower communities in preventing the spread of COVID-19. These guidelines were initially focused on preventive behaviors and later modified to include the promotion of vaccination at the community level upon the availability of the COVID-19 vaccine. However, the MSGs could also adopt other community-level strategies for mitigating the spread of the disease.

The outcomes of the activities of the MSGs are usually assessed by physical district reviews conducted by a team of HPB experts that visit each district individually. However, due to COVID-19 restrictions, this was not performed. Instead, the performance of each district and adherence to strategies were monitored through monthly virtual presentations. In addition, the case numbers and new clusters of cases were separately monitored in each district by the Epidemiology Unit within the Ministry of Health. The HPB was also part of this monitoring mechanism.^15^ Nevertheless, the HPB did not have data for the adaptation of the strategies by each MSG. Therefore, toward the end of 2021, each district was asked to select the best MSG conducting COVID-19 prevention activities in communities among all MSGs in their districts and send the selected MSG to the HPB.

The HPB initially linked MSGs and other platforms at the village and district levels, such as community support organizations and religious leaders.^16^ Most of the presented activities were conceived of and initiated by MSG members. The MSGs chose the best activities for their communities based on problem tree analysis methodology. All MSG members received routine training on this from the preventive health staff of the area. Some MSG members received specific technical inputs from the PHM and other preventive health staff, such as the public health inspector (PHI). The public health officers only acted as facilitators. Furthermore, religious leaders and government officials of the area also helped with some of the activities. The MSG members themselves generated funds for these activities. In some regions, donors also helped the members of MSGs by providing essential goods for quarantined people.

The HPB organized a national review in December 2021, and each MSG was asked to present its success stories on December 9–10, 2021. Each district team presented activities conducted by the MSG in the districts. A sample of 26 PHMs was selected to give presentations for the virtual event. The sample was purposive. The entire event was recorded, and the video recordings were transcribed using Microsoft Word by 2 coders. No special software was used for the analysis. We did not quantify the interpretations of the respondents, and the researchers noted their own influence on data to minimize bias. The qualitative data analysis process was data-driven and inductive.^17^ A thematic analysis was done using pattern recognition, with emerging themes becoming the categories for analysis. The following key themes were identified from the presented content with barriers and facilitating factors.^18^

- Establishment of communication networks
- Creation of a supportive environment for preventive behaviors
- Organization of COVID-19 vaccination clinics
- Distribution of essential food items to people in need
- Distribution of routine clinic medicines to patients with noncommunicable diseases (NCDs)
- Organization of recreational activities for children and adults
- Promotion of home gardening
- Monitoring and evaluation of the work

### Establishment of Communication Networks

In adapting to the “new normal” era, community empowerment required changing old patterns and making new ones. Meeting with the community in person was limited, and new modalities of communicating with the community members were established.^19^ Similarly, most of the MSGs have created new mechanisms to communicate with their members and their communities. The majority of them selected the WhatsApp platform for sharing information, education, and communication materials and other related documents on COVID-19. In addition, some MSG members used a public address system to raise awareness and distribute relevant messages. At the same time, some of the MSG members used existing notice boards in public places to paste notices and announcements related to COVID-19 prevention.

### Creation of a Supportive Environment for COVID-Appropriate Behaviors

One of the key strategies identified in health promotion is the creation of supportive environments.^5^ These environments can be implemented at the community level to promote behaviors, such as handwashing, mask-wearing, social distancing, and regularly cleaning surfaces. MSG members had created many innovative, supportive environments to practice preventive behaviors using minimal resources, such as handwashing facilities consisting of a bucket with a tap mounted on it in front of shops, supermarkets, and houses. Other activities included marking 1-meter footsteps for social distancing in supermarkets and at automated teller machines.

### Organization of COVID-19 Vaccination Clinics

COVID-19 vaccines provided hope for ending the pandemic with the assumptions of equal access and optimal uptake worldwide.^20^ However, the vaccine acceptance rate in Sri Lanka was initially predicted to be moderate.^21^ Therefore, to increase vaccine acceptance in the community, MSGs members played an active role in helping to arrange clinic settings and alleviate people’s fear regarding vaccination. In addition, members of MSGs used their communication networks to inform people in their community of the dates of vaccination clinics and the importance of vaccination.

### Distribution of Essential Food Items to People in Need

The provision of emergency aid has been identified as one of the major activities coordinated through religious leaders and institutions in the community in Sri Lanka.^16^ Some MSGs distributed essential food items to those in their community who lost their occupations due to COVID-19 and those with difficulty accessing essential food items, especially lone elders and people with disabilities. The members of the MSGs collected the necessary items for food packs, which were distributed to vulnerable people in the communities. Most community members who could afford it willingly contributed and appreciated the group’s effort.

### Distribution of Routine Clinic Medicines to Patients With NCDs

**Figure 1:**
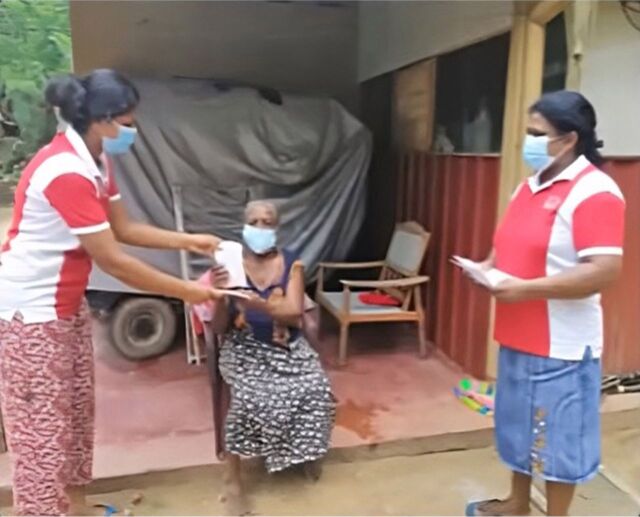
Two mothers’ support group members deliver routine clinic medicines to an elderly patient in Sri Lanka.

The COVID-19 pandemic resulted in a widespread NCD health service disruption that placed enormous strain on health systems in the South East Asia region.^22^ The health sector sought many innovative solutions in Sri Lanka to facilitate medication delivery through major hospitals.^23^ However, public transport buses were not functioning due to the quarantine curfew. Therefore, most people in the communities who used public transport services were affected, especially those traveling for regular clinic visits. They had to use alternative transport methods to visit the hospital clinics, and some people could not afford a taxi. MSG members initiated a novel arrangement by which they collected the clinic books of these patients and procured the relevant medicines from local hospitals. Usually, patients with chronic NCDs who wish to get treated in government hospitals are registered in the government hospital clinic in their area. A clinic book is maintained for recording the patient’s clinical history, examination, investigation findings, and the routine medicines prescribed by the clinic. During the COVID-19 pandemic, routine NCD medications, which the hospital provided for 1 month, were dispatched to certain patients with the help of the MSG members, according to the last record of the clinic book. The local hospitals supported this initiative in many districts.

MSG members procured medicines from hospitals for patients with NCDs who were unable to travel during quarantine.

### Organization of Recreational Activities for Children and Adults

COVID-19 affected the psychosocial well-being of many communities. Recent evidence highlights increased mental health problems such as depression and anxiety. Therefore, many recommendations emphasize the importance of allocating time for hobbies and leisure activities, while adhering to COVID-19 guidelines.^24^ Some MSG members have engaged in organizing art competitions for children, and winners were awarded while adhering to national COVID-19 guidelines. Furthermore, some MSG members organized musical shows via Zoom, which were very popular among communities.

### Promotion of Home Gardening

Home gardening promotion during the pandemic was identified as an intervention to improve self-reported physical and mental well-being^25^ with economic benefit.^26^ Therefore, MSG members encouraged home gardening in the communities during the quarantine curfew. Furthermore, MSG members distributed the seeds necessary for home gardening and arranged advice through the agricultural officer allocated for that area. Moreover, they encouraged sharing the crop gains from the home garden among the villagers.

### Monitoring of Community-Level Activities

One of the key recommendations for COVID-19 prevention and control through community engagement approaches^5^ is to incorporate community members into the planning process and response and monitoring of standard operating procedures. Community remote monitoring and alert systems are considered vital components of this activity. MSGs have engaged in the monitoring of preventive behaviors, quarantine procedures, and contact tracing activities. MSG members were voluntarily monitoring adherence to quarantine procedures among their villagers and the practice of some preventive behaviors, such as handwashing practices and maintenance of 1 meter in front of the shops, supermarkets, and automated teller machines in their area. Area PHMs and PHIs were alerted via local social media groups of the shortcomings of these practices. The members of MSGs were also involved in monitoring the contact tracing activities supporting the PHIs. Furthermore, MSGs were also involved in monitoring vaccine uptake in some areas. In areas where the vaccine uptake was low, the MSGs informed the PHI about the households that were eligible to receive vaccines during the various vaccination campaign phases. In addition, some of the groups have encouraged the creation of a COVID-19 calendar for affected families. These calendars helped the affected families to monitor their disease at the household level.

## LESSONS LEARNED: BARRIERS AND FACILITATING FACTORS

Communities were severely affected during the COVID-19 pandemic and were required to adapt to sudden changes that restricted their freedom; this impacted the communities' potential welfare, leading to a new normal way of living.^19^ Community engagement was one of the key interventions used by many countries to control the pandemic.^5^

Sri Lanka faced 3 prominent waves of COVID-19 (3,396 cases and 13 deaths in the first wave; 92,341 cases and 591 deaths in the second wave; and 163,352 cases and 2,473 deaths in the third wave).^27^ The first wave in Sri Lanka was mainly controlled through strict adherence to preventive behaviors.^28^^,^^29^ Once the vaccines were introduced, combining vaccination with other preventive behaviors was considered the mainstay of prevention and control of COVID-19. MSGs directly participated in many of the preventive activities conducted by the health authorities at the village level. As described above, the MSGs have employed many successful community-level methods in overcoming the COVID-pandemic in Sri Lanka.

The MSGs have employed many successful community-level methods in overcoming the COVID-19 pandemic in Sri Lanka.

We have adapted the themes described by Harden et al.^30^ in discussing barriers and facilitators of community engagement initiatives practiced by the MSGs. These themes include: (1) the quality of existing relationships with the communities; (2) organizational culture; (3) attitudes and practice; (4) investment in infrastructure and planning to support community engagement; (5) support, training, and capacity-building; (6) capabilities and the engagement process; and (7) an inclusive and accessible approach.

Regarding the quality of existing relationships with the communities, the MSGs were well recognized and villages did not see them as a threat. However, the organizational commitment was at a suboptimal level, with resistance from PHMs and PHIs to share power. Even though the MSG members are empowered in health and well-being activities, PHMs and PHIs primarily provide preventive health services. Therefore, the MSG members mainly play a supportive role in these activities. However, this was challenged during the pandemic, when the PHMs and PHIs needed extensive support from community groups in conducting control activities. Reluctance of these workers to share power with the community groups was identified as a major barrier by many MSGs. Furthermore, since the MSGs fall under the volunteer category, purely working without attachment to local or regional health institutions, many regional health authorities were not committed to their welfare. Therefore, many effective activities conducted by the MSGs were not appreciated or recognized by the local or regional level health authorities. This lack of appreciation and recognition was a major barrier for MSGs in obtaining support from other non-health sector partners.

The supportive culture and attitudes that were present at the beginning of the pandemic were lacking later on, which may be due to “pandemic fatigue”^31^ and a reduction in perceived susceptibility to the disease^32^ toward the end. Regarding investment in infrastructure and planning, there were limited health staff available to facilitate the community engagement process of the MSGs, and competing agendas across stakeholders were major barriers.

However, there were several facilitating factors, such as many initiatives in the communities that were planned well with a joint decision-making process. Furthermore, the MSGs received support from existing partnerships and networks, such as religious leaders and community influencers.^16^ Under the support, training, and capacity-building theme, the MSGs and communities regularly received support and training from the district health education officers network. However, a lack of capacity and difficulties in engaging vulnerable groups were noted during the early stages of the pandemic. Nevertheless, gaining direct access to the communities as a result of recognizing the use of MSGs as a community empowerment approach was a facilitating factor. This was also due to the longstanding nature and trust of the communities since the MSGs were well established and were supported by PHMs and PHIs. Geographic boundaries and cultural and language issues were not identified as significant barriers due to reasons such as MSG members being from the same communities.

Although there is limited literature on COVID-19 community engagements of similar nature (as we have described with MSGs), approaches comparable to the MSG system in Sri Lanka have been documented in many other countries for strengthening maternal and child health. These are called the “Care Groups,” which were developed to support maternal and child health in resource-constrained settings.^33^ The notable difference between the 2 approaches is that in Care Groups, some of the staff are paid (by NGOs), and there is minimum involvement of ministries of health. In contrast, no incentives are paid in MSGs, and public health workers are involved.^33^ However, the Care Group approach has been shown to be effective in reducing childhood malnutrition and mortality in children younger than 5 years.^34^ Therefore, similar community engagement interventions we have observed can be replicated using these Care Groups.

It is also worth noting that the community engagement initiatives undertaken by the MSGs in Sri Lanka further strengthen the evidence linking the concept of social capital with low mortality, better self-rated health, and the practice of healthy behaviors.^35^ The success of the MSGs at the community level can be explained by notions of social capital, such as the overall improvement in access to financial and social resources and reliable social relationships leading to better health outcomes.^36^ In addition, it can be explained in the form of “bonding social capital,” which refers to social connections between socially similar individuals (e.g., MSG members and other mothers in the communities that were affected), and in the form of “bridging social capital,” which refers to connections between socially dissimilar individuals (e.g., MSG members and other hierarchical health and non-health stakeholders).^37^

The success of the MSG initiatives strengthens evidence linking social capital with low mortality, better self-rated health, and the practice of healthy behaviors.

## CONCLUSIONS

Community engagement initiatives can play a crucial role during disease outbreak control activities. The community-level methods employed by MSGs in Sri Lanka ranged from promoting preventive behaviors to organizing simple recreational activities. Our review showed that adaptation to the sociocultural context of the particular community is important to improve efficiency and overall success in community engagement. Furthermore, it revealed the importance of the continuous development of the knowledge and skills of MSG members, appreciation of MSG activities by the health authorities, and promotion of power-sharing among partners for successful community engagement.

## Notes

**Peer Reviewed**

**First published online:** April 20, 2023.
